# Value of Computerized 3D Shape Analysis in Differentiating Encapsulated from Invasive Thymomas

**DOI:** 10.1371/journal.pone.0126175

**Published:** 2015-05-04

**Authors:** Jong Hyuk Lee, Chang Min Park, Sang Joon Park, Jae Seok Bae, Sang Min Lee, Jin Mo Goo

**Affiliations:** 1 Department of Radiology, Seoul National University College of Medicine, and Institute of Radiation Medicine, Seoul National University Medical Research Center, Seoul, Korea; 2 Cancer Research Institute, Seoul National University, Seoul, Korea; University of Groningen, University Medical Center Groningen, NETHERLANDS

## Abstract

**Objectives:**

To retrospectively investigate the added value of quantitative 3D shape analysis in differentiating encapsulated from invasive thymomas.

**Materials and Methods:**

From February 2002 to October 2013, 53 patients (25 men and 28 women; mean age, 53.94 ± 13.13 years) with 53 pathologically-confirmed thymomas underwent preoperative chest CT scans (slice thicknesses ≤ 2.5 mm). Twenty-three tumors were encapsulated thymomas and 30 were invasive thymomas. Their clinical and CT characteristics were evaluated. In addition, each thymoma was manually-segmented from surrounding structures, and their 3D shape features were assessed using an in-house developed software program. To evaluate the added value of 3D shape features in differentiating encapsulated from invasive thymomas, logistic regression analysis and receiver-operating characteristics curve (ROC) analysis were performed.

**Results:**

Significant differences were observed between encapsulated and invasive thymomas, in terms of cystic changes (*p*=0.004), sphericity (*p*=0.016), and discrete compactness (*p*=0.001). Subsequent binary logistic regression analysis revealed that absence of cystic change (adjusted odds ratio (OR) = 6.636; *p*=0.015) and higher discrete compactness (OR = 77.775; *p*=0.012) were significant differentiators of encapsulated from invasive thymomas. ROC analyses revealed that the addition of 3D shape analysis to clinical and CT features (AUC, 0.955; 95% CI, 0.935–0.975) provided significantly higher performance in differentiating encapsulated from invasive thymomas than clinical and CT features (AUC, 0.666; 95% CI, 0.626–0.707) (*p*<0.001).

**Conclusion:**

Addition of 3D shape analysis, particularly discrete compactness, can improve differentiation of encapsulated thymomas from invasive thymomas.

## Introduction

Thymomas originate from epithelial cells of the thymus [1], and are the most common primary neoplasm in the anterior mediastinum, representing 47–50% of all anterior mediastinal tumors [2–4]. For tumor staging of thymomas, Masaoka-Koga staging is most commonly used since it has been shown to be well correlated with patients’ survival. Accordingly, treatment plans have also generally been determined using this staging system [5]. The Masaoka-Koga staging system is based on gross and microscopic invasion of thymomas into adjacent structures, i.e., stage I tumors designate completely encapsulated tumors; stage II tumors show only microscopic invasion into capsules (IIa) or macroscopic invasion into perithymic fat (IIb); and stage III tumors invade neighboring organs such as the pericardium, great vessel, or lung. Stage IV tumors show metastasis, which can be limited to pleural or pericardial dissemination (IVa) or lymphatic or hematogenous metastasis (IVb).

Pre-operative differentiation of encapsulated thymomas (Masaoka-Koga stage I) from invasive thymomas (equal to or greater than Masaoka-Koga stage II) has an important clinical significance in that it guarantees complete resection without potential seeding and allows surgical planning such as minimally invasive surgery. Moreover, it can help to predict excellent prognosis in patients with encapsulated thymomas. In fact, there have been many studies in which preoperative CT morphologies of thymomas could be used as differentiators of encapsulated from invasive thymomas, including tumor shape, irregularity of tumor contour, hemorrhage, necrosis, and cystic change [1, 2, 4–15]. However, despite of the usefulness of these morphologic features, there exists primary concern regarding visually-determined features owing to potential interpretation variability between observers and even within the same observer.

In this context, computer-aided quantitative three-dimensional (3D) shape analysis can be a promising differentiating tool as it can provide a more detailed and, importantly, reproducible quantitative assessment of lesion characteristics compared to visual analysis by human observers. To our knowledge, however, there have been no studies evaluating the usefulness of quantitative 3D shape features in differentiating encapsulated from invasive thymomas. Thus, the purpose of this study was to investigate the added value of quantitative 3D shape analysis to clinical and CT features in differentiating encapsulated from invasive thymomas.

## Subjects and Materials and Methods

This retrospective study was approved by the institutional review board of Seoul National University Hospital (IRB number: 1406-008-585) and the requirement for informed consents was waived.

### Study population

One author (J.H.L.) searched the electronic medical records and the radiology information systems of our hospital, and found 255 patients who had undergone surgical resection for thymomas in our institution between February 2002 and October 2013. We selected all cases that met the following criteria: 1) Patients with pathologically-confirmed thymomas via surgical resection; 2) patients without a history of chemotherapy or radiation therapy prior to surgical resection of thymomas; and 3) patients who had available preoperative thin-slice chest CT images with a slice thickness ≤ 2.5 mm. A total of 202 patients were excluded due to the following reasons: Patients who had 1) preoperative chest CT for thymoma with a slice thickness >2.5mm (n = 123) or 2) history of neoadjuvant chemotherapy or radiation therapy (n = 62) or 3) surgical resection at outside hospital without detailed surgical or pathological reports (n = 17). Finally, a total of 53 patients (mean age, 53.94 ± 13.13 years; range, 22–84 years) with 53 thymomas were included in this study. Among these 53 patients, 25 were men (mean age, 54.96 ± 14.35 years; range, 22–84 years) and 28 were women (mean age, 53.04 ± 12.13 years; range, 23–74 years). As for thymoma staging, 23 (43.4%) were pathologically confirmed as encapsulated thymomas and 30 (56.6%) as invasive thymomas. Among the 30 invasive thymomas, 26 were determined to be Masaoka-Koga stage II (49.1%) and 4 as stage IV (7.5%). Among the 53 patients, three patients had type A thymomas (5.7%), 10 type AB (18.9%), 22 type B1 (41.5%), 11 type B2 (20.7%) and 7 had type B3 (13.2%) according to the WHO histological classification of thymoma [5, 11–12].

### CT technique and image acquisition

Forty-nine patients underwent preoperative CT scans with 75–90 ml of nonionic iodinated contrast material (370 mg I/mL Iopromide; Ultravist 370; Bayer Shering Pharma AG, Berlin, Germany), while 4 underwent preoperative CT scans without contrast material. All CT examinations were performed using one of six available CT scanners: Sensation-16, Somatom Definition (Siemens Medical Systems, Erlangen, Germany), Brilliance-64, Ingenuity (Phillips Medical Systems, Best, The Netherlands), and Discovery CT750 HD, LightSpeed Ultra (GE Healthcare, Milwaukee, WI, USA). Scanning parameters for chest CT were as follows: detector collimation, 1.0–1.25 mm; beam pitch, 0.75–1.0; reconstruction slice thickness, 0.7–2.5 mm (0.7 mm (n = 1), 1.0 mm (n = 28), 1.25 mm (n = 22), 1.5 mm (n = 1), 2.5 mm (n = 1)); reconstruction interval, 1.0–1.25 mm; rotation time, 0.4–0.5 s; tube voltage, 120 kVp; tube current, 40–120 mAs; and reconstruction kernel, a sharp reconstruction algorithm. All CT scans were performed during patients’ inspiration in the supine position.

The mean time interval between preoperative CT scans and surgical resections was 13.91 ± 13.63 days (range, 1–58 days).

### Image interpretation and computerized 3D shape analysis

**Fig 1:**
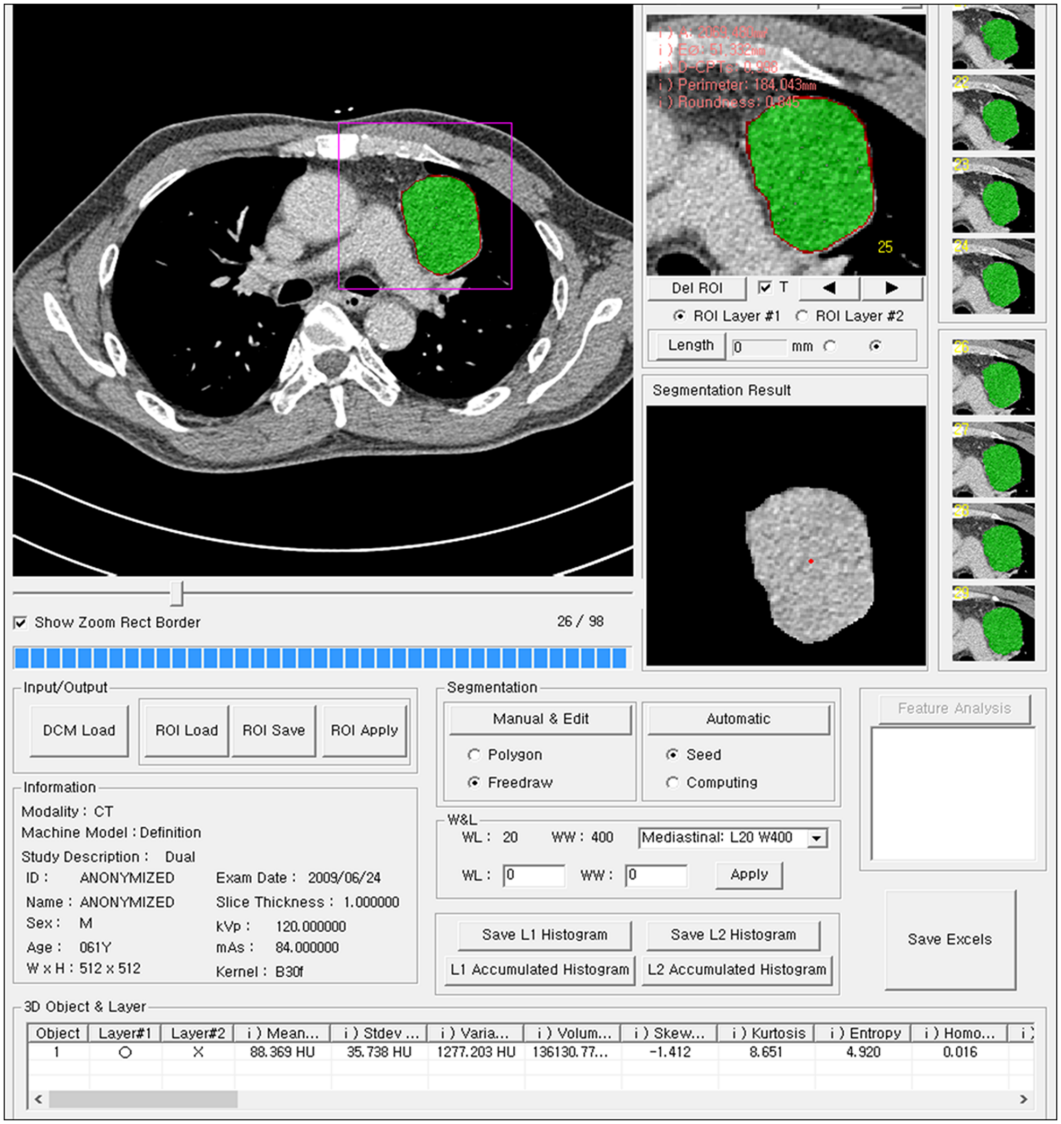
3D shape analysis software program. Each thymoma was manually segmented from surrounding structures on all CT images and their 3D shape features were automatically calculated using an in-house developed software program.

Characteristic CT image features of thymomas were reviewed in terms of tumor size, cystic change, and calcification. Cystic change was designated when there were definite focal circumscribed areas of low attenuation within tumors on CTs [11, 12]. In addition, each thymoma was manually segmented from surrounding structures on all CT images containing tumors, and their 3D shape features were automatically calculated using an in-house developed software program (Fig 1) [16]. Manual segmentation of the 53 thymomas was performed by one radiologist (J. H. L with 2 years of experience in chest CT) and confirmed by one chest radiologist (C. M. P. with 15 years of experience in chest CT). Quantitative 3D shape features included: (a) log_volume, (b) surface area (cm^2^), (c) sphericity, (d) discrete compactness, and (e) 3D roundness. We used natural logarithms (log_volume) of tumor volume instead of tumor volume as the data of natural logarithms of tumor volume follows a normal distribution according to the Kolmogorov-Smirnov test. The formulas as well as further detailed explanation of these 3D shape features are provided in S1 File.

For inter-observer variability, 16 thymomas were randomly selected. The sample size calculation in this variability test was based on the ability to establish correlation coefficient of at least 0.60 (probability of type I error (α), 0.05; power, 0.8). Thereafter, another radiologist (J. S. B. with 2 years of experience in chest CT) independently performed manual segmentation of the selected 16 thymomas (8 encapsulated and 8 invasive thymomas) for this inter-observer test. Finally, inter-observer variability of 3D shape features obtained with manual segmentation was investigated using intraclass correlation coefficients (ICC) (agreement) [17–20]. ICC of < 0.40, signified poor agreement; 0.41–0.60, moderate agreement; 0.61–0.80, good agreement; and 0.81 or greater, excellent agreement [21].

### Statistical analyses

To identify significant differentiating variables between encapsulated and invasive thymomas, univariate analyses were performed using Pearson's chi-square test, Fisher's exact test and Student's t-test, as appropriate. Subsequent binary logistic regression analysis was performed to evaluate independent differentiating factors. In total, three separate logistic regressions were performed: 1) clinical and CT features alone, 2) 3D shape features alone, and 3) combination of clinical, CT and 3D shape features. In binary logistic regression analysis, variables with a P-value < 0.1 at univariate analysis were used as input variables for each model, and backward stepwise selection mode was employed with iterative entry of variables based on test results (*p* < 0.05). The removal of variables was based on likelihood ratio statistics with a probability of 0.10.

Three binary logistic regression models using backward stepwise selection mode were used to build a differentiating models of encapsulated from invasive thymomas. Significant differentiators were used as input data for these three models, respectively. We utilized the leave-one-out cross-validation method to train and test our binary logistic regression models. The leave-one-out cross-validation method is an extreme form of the k-fold cross-validation method, and all subjects except one are used as the training set and excepted one is used for testing set for the model. The algorithm continues iteratively with each subject in the cohort until all subjects are used exactly once for testing [21]. Receiver operating characteristic curve (ROC) analysis [22] were performed to determine the differentiating performance of three established logistic regression models, and their differentiating performances were compared to assess the significant added value of 3D shape features in differentiating encapsulated from invasive thymomas.

All statistical analyses were performed using SPSS ver. 20.0 (SPSS Inc., Chicago, IL, USA), MedCalc ver. 12.0 (MedCalc Software, Mariakerke, Belgium), and STATA ver. 12.0 (College Station, Tex).

## Results

### Patient and Disease Characteristics

Among the 53 patients, 11 patients (2 with encapsulated thymomas; 9 with invasive thymomas) complained of symptoms such as chest pain and cough, and 11 patients (4 with encapsulated thymomas; 7 with invasive thymomas) were diagnosed with myasthenia gravis.

### Analysis of CT features and 3D shape analysis

Nineteen thymomas showed cystic change (3 encapsulated thymomas; 16 invasive thymomas) and 11 thymomas showed internal calcification (4 encapsulated thymomas; 7 invasive thymomas).

**Fig 2:**
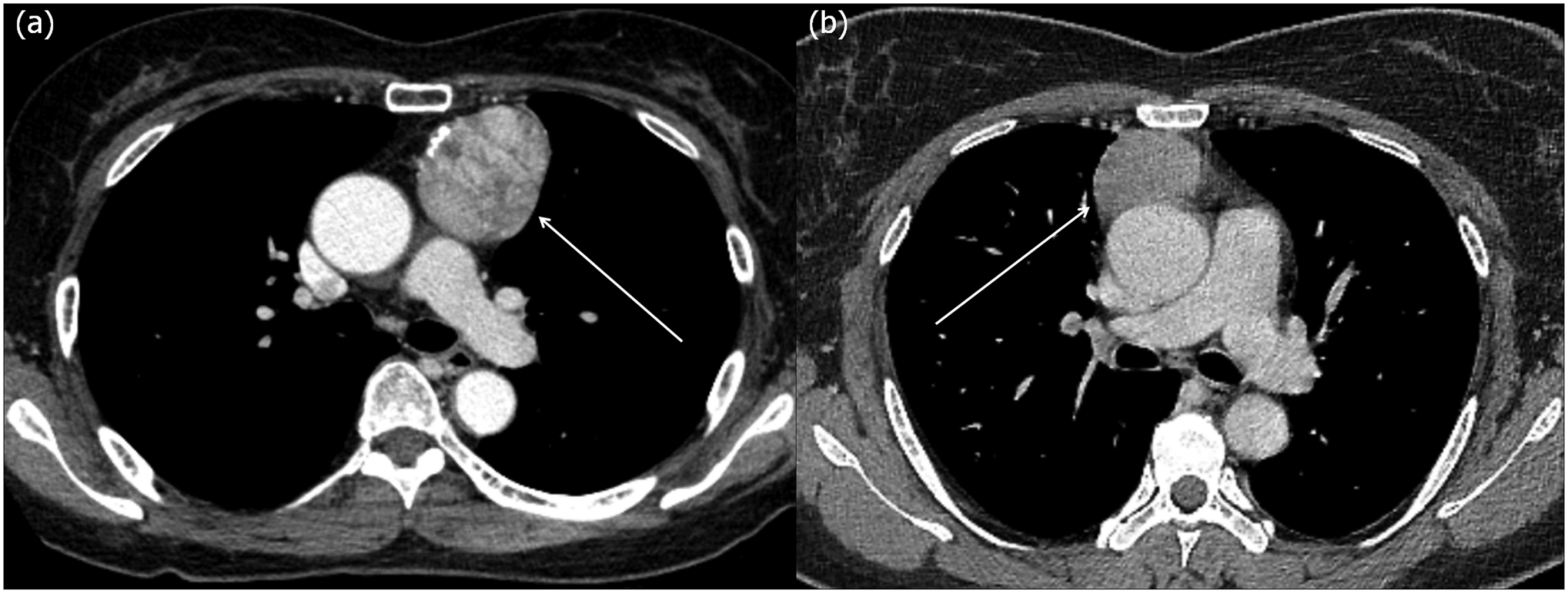
CT images of encapsulated and invasive thymomas. (a) A 61 year old female who underwent surgical resection of an encapsulated thymoma (arrow) (discrete compactness, 0.925; sphericity, 0.703). (b) A 40 year old female who underwent surgical resection of an invasive thymoma (arrow) (discrete compactness, 0.722; sphericity, 0.646). Note that although these two kinds of thymomas cannot be easily differentiated grossly owing to similar CT features, there is a distinct difference in 3D shape features, particularly in discrete compactness, between the encapsulated thymoma and invasive thymoma.

On univariate analysis, significant differences were observed in cystic change (encapsulated thymomas, 3/23; invasive thymomas, 16/30; *p* = 0.004), sphericity (0.677 vs. 0.604; *p* = 0.016), and discrete compactness (0.825 vs. 0.691; *p* = 0.001). The results of univariate analysis discriminating encapsulated from invasive thymomas are summarized in Tables 1 and 2 (Fig 2).

**Table 1:** Univariate analysis of clinical and CT features of encapsulated and invasive thymomas.

Variable	Encapsulated thymomas	Invasive thymomas	P-value
Age (year)	52.0 ± 12.7	55.3 ± 13.6	0.368
Presence of symptoms	2	9	0.089
Presence of myasthenia gravis	4	7	0.738
Presence of cystic change	3	16	0.004
Presence of calcification	4	7	0.738
Diameter (cm)	4.9 ± 1.7 (range, 2.1–7.8)	4.2 ± 1.8 (range, 1.7–9.1)	0.187
WHO	A (n = 1)	A (n = 2)	0.325
classification	AB (n = 7)	AB (n = 3)	
	B1 (n = 10)	B1 (n = 12)	
	B2 (n = 3)	B2 (n = 8)	
	B3 (n = 2)	B3 (n = 5)	

Note—Data are numbers or mean ± standard deviation of each variable.

**Table 2:** Univariate analysis of 3D shape features of encapsulated and invasive thymomas.

Variable	Encapsulated thymomas	Invasive thymomas	P-value
Log_Volume	1.631 ± 0.529 (range, 0.879–3.201)	1.335 ± 0.604 (range, 0.402–3.076)	0.530
Surface area (cm^2^)	89.989 ± 59.312 (range, 25.773–248.384)	83.552 ± 86.609 (range, 11.469–452.633)	0.761
Sphericity	0.677 ± 0.106 (range, 0.48–0.81)	0.604 ± 0.104 (range, 0.434–0.795)	0.016
Discrete compactness	0.825 ± 0.106 (range, 0.472–0.928)	0.691 ± 0.160 (range, 0.37–0.900)	0.001
Roundness	0.699 ± 0.068 (range, 0.546–0.811)	0.685 ± 0.074 (range, 0.569–0.818)	0.486

Note—Data are mean ± standard deviation.

Subsequent binary logistic regression analysis with all significant input variables of clinical, CT features, and 3D shape revealed that absence of cystic change (adjusted odds ratio (OR) = 6.636; *p* = 0.015) and higher discrete compactness (OR = 77.775; *p* = 0.012) were significant discriminating factors of encapsulated from invasive thymomas (Table 3). As for multi-collinearity, there were no variables which showed variance inflator factor (VIF) more than 10.

**Table 3:** Binary logistic regression analysis in differentiating encapsulated from invasive thymomas.

Models	Significant features	Adjusted OR†	95% CI††	P-value
Clinical and CT features, alone	Cystic change	7.619	1.861–31.196	0.005
3D shape features, alone	Discrete compactness	92.110	3.828–2216.460	0.005
Clinical and CT features + 3D shape features	Cystic change	6.636	1.452–30.335	0.015
	Discrete compactness	77.775	2.595–2331.333	0.012

^†^OR = odds ratio

^††^CI = confidence interval

**Fig 3:**
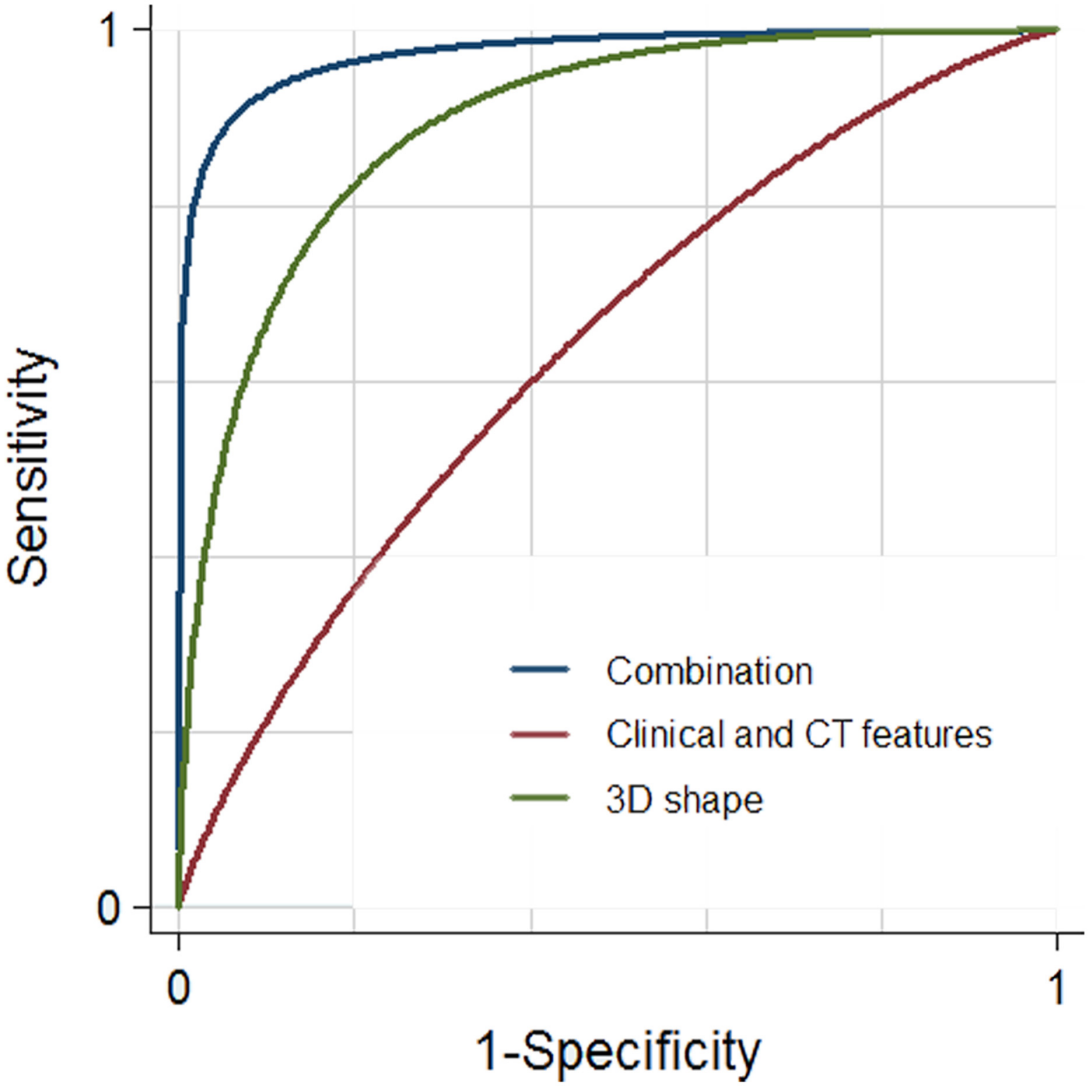
ROC plot of binary logistic regression analysis with backward stepwise selection, using leave-one-out cross-validation method. Receiver operating characteristics (ROC) curve analysis of binary logistic regression models, using leave-one-out cross-validation method, in differentiating encapsulated from invasive thymomas. The graph shows that the combination of 3D shape analysis and CT features (blue line, AUC, 0.955; 95% CI, 0.935–0.975) has significantly higher discriminating performance in differentiating encapsulated from invasive thymomas compared to clinical and CT features (red line, AUC, 0.666; 95% CI, 0.626–0.707) (difference between AUC values, 0.289; *p*<0.001). For reference, ROC analysis with 3D shape analysis alone is also demonstrated (green line, AUC, 0.896; 95% CI,0.868–0.923).

Finally, ROC analyses revealed that the addition of 3D shape analysis to CT features (AUC, 0.955; 95% CI, 0.935–0.975) provided significantly higher discriminating performance than clinical and CT features alone (AUC, 0.666; 95% CI, 0.626–0.707) (difference between AUC values, 0.289; *p* < 0.001). For reference, ROC analysis with 3D shape analysis alone showed an AUC of 0.896 (95% CI, 0.868–0.923) (Fig 3).

### Inter-observer variability analysis of 3D shape features

The results of inter-observer variability for 3D shape feature analysis between the two radiologists are presented in Table 4. All 3D shape features calculated from two different sets of ROIs independently segmented by two radiologists showed excellent agreement (ICC range, 0.936–0.994).

**Table 4:** Interobserver variability of shape features of thymomas.

Variables	ICC†	95% CI††	P-value
Log_Volume	0.994	0.983–0.998	<0.001
Surface area (cm^2^)	0.984	0.955–0.994	<0.001
Sphericity	0.936	0.816–0.978	<0.001
Discrete compactness	0.969	0.911–0.989	<0.001
Roundness	0.992	0.978–0.997	<0.001

Note—ICCs of less than 0.40 signifies poor agreement; 0.41–0.60, moderate agreement; 0.61–0.80, good agreement; and 0.81 or greater, excellent agreement.

^†^ICC = intraclass correlation coefficients.

^††^CI = confidence interval

## Discussion

The Masaoka-Koga staging system is based on the degree of thymomas’ invasiveness and has been widely used since it has been reported to be well correlated with prognosis and utilized to dictate treatment planning [5]. There have been many attempts to identify variables able to differentiate encapsulated from invasive thymomas prior to treatment in the radiologic field [1, 10, 13]. Tomiyama, et al. [10] reported CT features of thymomas in 50 patients and suggested that invasive thymomas were more likely to have a cystic or necrotic portion, calcification, a lobulated or irregular contour and larger size than encapsulated thymomas. Priola, et al. [13] also reported that invasive thymomas had significantly larger size, more lobulated or irregular contours, cystic or necrotic portions, and foci of calcifications than encapsulated thymomas. These two studies also suggested that partial or complete obliteration of fat planes around the tumor was not helpful in distinguishing invasive from encapsulated thymomas. In another study, Qu, et al. [1] suggested that the invasiveness of thymomas were significantly associated with variables such as tumor size, irregular shape, uneven density, incompleteness of capsules, or involvement of surrounding tissues. However, visual shape analysis including that of the tumor contour is subjective and thus susceptible to inter or intra-observer interpretation variability, warranting a need for an objective and reproducible method in evaluating the shape of thymomas.

In the present study, we adopted computerized 3D shape features such as sphericity, discrete compactness and roundness of thymomas and found that encapsulated thymomas showed significantly different values in terms of sphericity (*p* = 0.016) and discrete compactness (*p* = 0.001) as well as cystic change (*p* = 0.004) from invasive thymomas. Subsequent binary logistic regression analysis confirmed that higher discrete compactness (OR = 77.775; *p* = 0.012) as well as the absence of cystic change (OR = 6.636; *p* = 0.015) were independent discriminators of encapsulated from invasive thymomas. In addition, ROC analyses revealed that a combination of 3D shape features and conventional clinical and CT features significantly enhanced the discriminating performance of encapsulated from invasive thymomas (AUC, 0.955; 95% CI, 0.935–0.975) compared with clinical and CT features alone (AUC, 0.666; 95% CI, 0.626–0.707) (difference between AUC values, 0.289; *p* < 0.001).

The discrete compactness of an object is defined as the ratio between the actual contact surface area and the theoretically calculated maximum contact surface area [23]. In other words, to obtain a constant value of volume for an object, an increased value of the actual contact surface of an object provides higher discrete compactness. Intuitively, the increased value of the actual contact surface is proportional to the circularity of the object, representing higher discrete compactness. Thus, it is not surprising that encapsulated thymomas have higher values of discrete compactness, compared with invasive thymomas, which tend to show lobulated or irregular contours more frequently than encapsulated thymomas owing to invasion beyond the tumor’s capsule. Indeed, Braumann et al. [16] suggested in their study that discrete compactness was correlated with the invasion of uterine cervical carcinoma.

Sphericity, on the other hand, did not turn out to be an independent differentiating feature of encapsulated thymomas from invasive thymomas according to binary logistic regression analysis even though encapsulated thymomas showed significantly higher sphericities than invasive thymomas on univariate analysis. As sphericity of the tumor can be correlated with contour irregularity or lobulation and it is known that invasive thymomas have significantly more lobulated or irregular contours [1, 10, 13], further research with a larger study population is warranted to determine the relationship between sphericity and the invasiveness of thymomas with high confidence. We also found that a combination of 3D shape features, particularly discrete compactness, and conventional clinical and CT features significantly enhanced the discriminating performance of encapsulated from invasive thymomas. With the use of this 3D shape feature, more precise preoperative staging may be accomplished and many clinicians can precisely distinguish patients who can have surgical resection without preoperative chemotherapy from patients who must undergo preoperative chemotherapy prior to surgical resection. In addition, with the precise diagnosis of encapsulated thymomas, minimally invasive surgery such as video-assisted thoracic surgery can be planned more confidently without concern of incomplete resection or pleural seeding.

We believe that standardization of image acquisition and maintenance of analyzable image quality, as well as selection of robust shape features, can be critical for this kind of computerized analytic method because it can be substantially influenced by image quality (e.g., spatial resolution, motion artifact due to respiration and heart pulsation, and image blurring) and various scan parameters. We believe that the utilization of raw imaging data without processing or normalization may be one of way overcoming obstacles related to variability and standardization issues [24].

Our study had several limitations. First, our study was of retrospective design, and thus was susceptible to potential selection bias. Second, the relatively small number of our study population might have limited the significance of other 3D shape features such as sphericity. Further studies with larger study populations are warranted to confirm our results. Thirdly, 3D shape feature analysis was performed, based on data from a number of different CT systems and image acquisition settings instead of uniformly controlled CT system and image acquisition setting. In this circumstance, the measured values of 3D shape feature analysis may be influenced by different scan parameters. Fourthly, the performance of our logistic regression model may be overly optimistic due to the potential over-fitting of model and lack of separate validation set, even though we utilized leave-one-out cross-validation method to overcome this limitation and to improve generalizability of the model. We hope further external validation studies could confirm our findings. Fifthly, 3D shape features in the present study were extracted from the results of manual segmentation by radiologists, which can be potentially influenced by observers’ subjective trends. However, fortunately, inter-observer variability tests in our study revealed that each 3D shape feature showed very excellent agreement between observers. Nevertheless, we believe that a reliable and robust automatic boundary extraction method can obviate this variability issue and enhance the clinical applicability of 3D shape analysis in daily clinical practice.

In conclusion, the addition of quantitative analysis of 3D shape features, particularly discrete compactness, to CT features provides higher predictive performance in the differentiation of encapsulated thymomas from invasive thymomas.

## Supporting Information

S1 File Formulas used to obtain quantitative 3D shape features. Tumor volume, Surface area, Sphericity, Discrete compactness, and 3D roundness.
(DOCX)

## References

[1] Qu Y-j, Liu G-b, Shi H-s, Liao M-y, Yang G-f, Tian Z-x. Preoperative CT findings of thymoma are correlated with postoperative Masaoka clinical stage. Acad Radiol. 2013;20(1):66–72. 10.1016/j.acra.2012.08.002 22981603

[2] Marom EM, Milito MA, Moran CA, Liu P, Correa AM, Kim ES, et al Computed tomography findings predicting invasiveness of thymoma. J Thorac Oncol. 2011;6(7):1274–1281. 10.1097/JTO.0b013e31821c4203 21623235

[3] Liu G-B, Qu Y-J, Liao M-Y, Hu H-J, Yang G-F, Zhou S-J. Relationship Between Computed Tomography Manifestations of Thymic Epithelial Tumors and the WHO Pathological Classification. Asian Pac J Cancer Prev. 2012;13(11):5581–5585. 23317221 10.7314/apjcp.2012.13.11.5581

[4] Ruffini E, Filosso PL, Mossetti C, Bruna MC, Novero D, Lista P, et al Thymoma: inter-relationships among World Health Organization histology, Masaoka staging and myasthenia gravis and their independent prognostic significan a single-centre experience. Eur J CardioThorac Surg. 2011;40(1):146–153. 10.1016/j.ejcts.2010.09.042 21093283

[5] Benveniste MF, Rosado-de-Christenson ML, Sabloff BS, Moran CA, Swisher SG, Marom EM. Role of imaging in the diagnosis, staging, and treatment of thymoma. Radiographics. 2011;31(7):1847–1861. 10.1148/rg.317115505 22084174

[6] Marom EM. Advances in thymoma imaging. J Thorac Imaging. 2013;28(2):69–83. 10.1097/RTI.0b013e31828609a0 23422781

[7] Marom EM. Imaging thymoma. J Thorac Oncol. 2010;5(10):S296–S303. 10.1097/JTO.0b013e3181f209ca 20859123

[8] Nishino M, Ashiku SK, Kocher ON, Thurer RL, Boiselle PM, Hatabu H. The Thymus: A Comprehensive Review. Radiographics. 2006;26(2):335–348. 16549602 10.1148/rg.262045213

[9] Sadohara J, Fujimoto K, Müller NL, Kato S, Takamori S, Ohkuma K, et al Thymic epithelial tumors: comparison of CT and MR imaging findings of low-risk thymomas, high-risk thymomas, and thymic carcinomas. Eur J Radiol. 2006;60(1):70–79. 16766154 10.1016/j.ejrad.2006.05.003

[10] Tomiyama N, Müller NL, Ellis SJ, Cleverley JR, Okumura M, Miyoshi S, et al Invasive and noninvasive thymoma: distinctive CT features. J Comput Assist Tomogr. 2001;25(3):388–393. 11351188 10.1097/00004728-200105000-00010

[11] Tomiyama N, Johkoh T, Mihara N, Honda O, Kozuka T, Koyama M, et al Using the World Health Organization Classification of thymic epithelial neoplasms to describe CT findings. AJR Am J Roentgenol. 2002;179(4):881–886. 12239030 10.2214/ajr.179.4.1790881

[12] Jeong YJ, Lee KS, Kim J, Shim YM, Han J, Kwon OJ. Does CT of thymic epithelial tumors enable us to differentiate histologic subtypes and predict prognosis? AJR Am J Roentgenol. 2004;183(2):283–289. 15269013 10.2214/ajr.183.2.1830283

[13] Priola A, Priola S, Di Franco M, Cataldi A, Durando S, Fava C. Computed tomography and thymoma: distinctive findings in invasive and noninvasive thymoma and predictive features of recurrence. Radiol Med. 2010;115(1):1–21. 10.1007/s11547-009-0478-3 20017005

[14] Takahashi K, Al-Janabi NJ. Computed tomography and magnetic resonance imaging of mediastinal tumors. J Magn Reson Imaging. 2010;32(6):1325–1339. 10.1002/jmri.22377 21105138

[15] Nasseri F, Eftekhari F. Clinical and Radiologic Review of the Normal and Abnormal Thymus: Pearls and Pitfalls. Radiographics. 2010;30(2):413–428. 10.1148/rg.302095131 20228326

[16] Braumann U-D, Kuska J-P, Einenkel J, Horn L-C, Höckel M. How to quantify cervical carcinoma invasion fronts in 3D. Bulletin. 2004;1:9–11.

[17] Bankier AA, Levine D, Halpern EF, Kressel HY. Consensus Interpretation in Imaging Research: Is There a Better Way? Radiology. 2010;257(1):14–17. 10.1148/radiol.10100252 20851935

[18] Lew R, Doros G. Design based on intra-class correlation coefficients. Am J Biostat. 2010;1(1):1.

[19] Landis JR, Koch GG. The measurement of observer agreement for categorical data. biometrics. 1977:159–174. 843571

[20] Shrout PE, Fleiss JL. Intraclass correlations: uses in assessing rater reliability. Psychol Bull. 1979;86(2):420 18839484 10.1037//0033-2909.86.2.420

[21] Chae H-D, Park CM, Park SJ, Lee SM, Kim KG, Goo JM. Computerized Texture Analysis of Persistent Part-Solid Ground-Glass Nodules: Differentiation of Preinvasive Lesions from Invasive Pulmonary Adenocarcinomas. Radiology. 2014;273(1):285–293. 10.1148/radiol.14132187 25102296

[22] DeLong ER, DeLong DM, Clarke-Pearson DL. Comparing the areas under two or more correlated receiver operating characteristic curves: a nonparametric approach. Biometrics. 1988:44(3):837–845. 3203132

[23] Bribiesca E. An easy measure of compactness for 2D and 3D shapes. Pattern Recognition. 2008;41(2):543–554.

[24] Aerts HJ, Velazquez ER, Leijenaar RT, Parmar C, Grossmann P, Carvalho S, et al Decoding tumour phenotype by noninvasive imaging using a quantitative radiomics approach. Nat Commun. 2014;5. 10.1038/ncomms5006 PMC4059926 24892406

