# Supplementary material for: Value of Computerized 3D Shape Analysis in Differentiating Encapsulated from Invasive Thymomas
Source: PLoS One. 2015 May 4;10(5):e0126175. doi: 10.1371/journal.pone.0126175 (PMC4418613; doi:10.1371/journal.pone.0126175)
Supplement: S1 File — Tumor volume, Surface area, Sphericity, Discrete compactness, and 3D roundness. (DOCX) [file pone.0126175.s001.docx]

**Supporting Information File 1.**

**Formulas used to obtain quantitative 3D shape features**

**Tumor volume (**$V$**)**

The *volume*, $V$ in $xyz$ coordinate is given by

$$V=N\cdot S_{x}\cdot S_{y}\cdot S_{z}$$

Where $N$ is the number of voxel in ROI, and $S$ is the spacing of the $xyz$ coordinate*.*

**Surface area (**$A_{C}$**)**

$$2A_{C}+A=aFn$$

Where $A_{C}$ is the contact surface area, $A$ is the area of the enclosing surface, $a$ is the area of the face of the polyhedron used, and $F$ is the number of faces of the polyhedron ($F$ is equal to 6 in case of voxels in digital domain).

**Sphericity** ($\psi$**)**

Sphericity is a measure of how spherical (round) an object is. As such, it is a specific example of the compactness measure of a shape. Defined by Wadell in 1935, the sphericity, $\psi$ is the ratio of the surface area of a sphere (with the same volume as the given mass) to the surface area of the mass:

$$\psi=\frac{\pi^{\frac{1}{3}}(6V)^{\frac{2}{3}}}{S_{A}}$$

Where  is the tumor volume, and is the surface area of the segmented tumor. The sphericity of a sphere is 1 and, through isoperimetric inequality, any shape which is not a sphere will have a sphericity of less than 1.


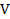

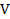

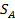

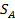


**Discrete compactness (**$C{}_{d}$**)**

Defined by Bribiesca in 2008, discrete compactness ($C{}_{d}$) is a computed measurement of any kind of object including porous and fragmented objects, defined as the ratio between the surface area and total surface voxel number in ROI.

Where $A_{C}$indicates the surface area, and $N$ is the number of voxels within the solid region in the ROI.

**3D roundness (angularity,** $R$**)**

3D Roundness, $R$ is the measure of how closely the shape of an object approaches that of a sphere. $R$ in $xyz$ coordinate is given by:

Where $N$ is the number of surface voxel in ROI, $R_{n}$ is defined as the distance between the center of mass and each surface point. indicates the average radius of the ROI. Typically, if the ROI close to sphere, the value $R$ becomes 1 (), or otherwise it close to 0.
